# Calcineurin inhibition prevents synaptic plasticity deficit induced by brain-derived tau oligomers

**DOI:** 10.1093/braincomms/fcae277

**Published:** 2024-08-16

**Authors:** Pietro Scaduto, Michela Marcatti, Nemil Bhatt, Rakez Kayed, Giulio Taglialatela

**Affiliations:** Department of Neurology, Mitchell Center for Neurodegenerative Diseases, University of Texas Medical Branch (UTMB), 301 University Blvd, Galveston, TX 77555, USA; Department of Neurology, Mitchell Center for Neurodegenerative Diseases, University of Texas Medical Branch (UTMB), 301 University Blvd, Galveston, TX 77555, USA; Department of Neurology, Mitchell Center for Neurodegenerative Diseases, University of Texas Medical Branch (UTMB), 301 University Blvd, Galveston, TX 77555, USA; Department of Neurology, Mitchell Center for Neurodegenerative Diseases, University of Texas Medical Branch (UTMB), 301 University Blvd, Galveston, TX 77555, USA; Department of Neurology, Mitchell Center for Neurodegenerative Diseases, University of Texas Medical Branch (UTMB), 301 University Blvd, Galveston, TX 77555, USA

**Keywords:** tacrolimus, FK506, tau, LTP, Alzheimer’s disease

## Abstract

Compelling evidence suggests that cognitive decline in Alzheimer’s disease is associated with the accumulation and aggregation of tau protein, with the most toxic aggregates being in the form of oligomers. This underscores the necessity for direct isolation and analysis of brain-derived tau oligomers from patients with Alzheimer’s disease, potentially offering novel perspectives into tau toxicity. Alzheimer’s brain-derived tau oligomers are potent inhibitors of synaptic plasticity; however, the involved mechanism is still not fully understood. We previously reported a significantly reduced incidence of Alzheimer’s disease in ageing humans chronically treated with a Food and Drug Administration–approved calcineurin inhibitor, FK506 (tacrolimus), used as an immunosuppressant after solid organ transplant. Using a combination of electrophysiological and RNA-sequencing techniques, we provide here evidence that FK506 has the potential to block the acute toxic effect of brain-derived tau oligomers on synaptic plasticity, as well as to restore the levels of some key synaptic mRNAs. These results further support FK506 as a promising novel therapeutic strategy for the treatment of Alzheimer’s disease.

## Teaser

Scaduto *et al*. report that tau oligomers, directly extracted from Alzheimer’s brain, cause synaptic impairment and consequent impairment of transcription induced after long-term potentiation. This toxicity is neutralized by FK506 (tacrolimus), a Food and Drug Administration–approved calcineurin inhibitor. These results suggest the central role of calcineurin in mediating tau toxicity.

## Graphical Abstract

**Graphical Abstract:**
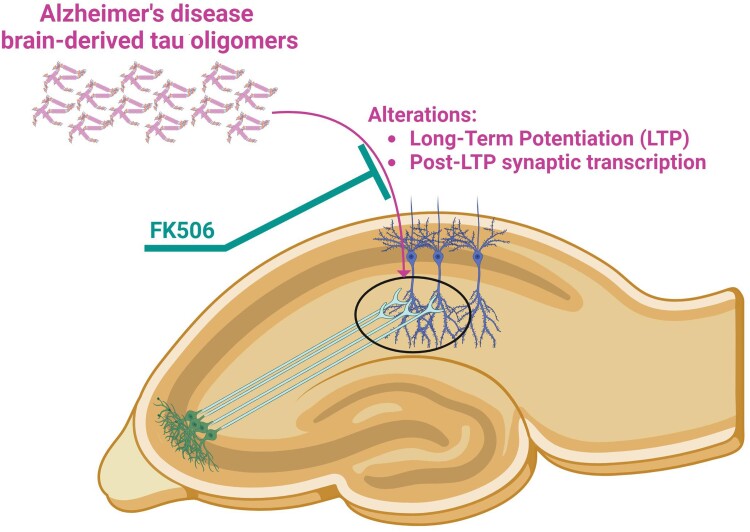

## Introduction

Alzheimer’s disease is the most prevalent form of neurodegenerative dementia. It manifests as memory deficits and impairments in various cognitive domains, including learning, language comprehension and visuospatial abilities.^1^ Alzheimer’s disease is a growing global health concern. Current treatments do not offer a resolving cure as their efficacy is limited by our incomplete understanding of the underlying pathophysiology.^2^ Hallmarks of Alzheimer’s disease include brain atrophy beginning in the entorhinal cortex and hippocampus, dysfunction and ultimately loss of neurons and synapses, and the deposition of β-amyloid (Aβ) and hyperphosphorylated tau proteins, which are observable as extracellular plaques and intracellular neurofibrillary tangles (NFTs), respectively.^3-6^ The severity of the pathology is categorized using Braak stages^7,8^ that quantify the distribution of hyperphosphorylated tau protein throughout the brain. This assessment is based on the reactivity of the AT8 antibody, which specifically binds to tau protein when phosphorylated at serine 202 and threonine 205. Together with dysfunctional synapses, Braak stage correlates best with cognitive decline.^9-12^ Present scientific data indicate that toxic tau species predominantly propagate in the brain in the form of soluble tau oligomers.^13^ These oligomers seem to exhibit greater toxicity compared with larger tau fibrils found in NFTs or tau monomers.^13,14^ Although many rigorous investigations have utilized recombinant tau (rTau), showing that it causes synaptic toxicity,^15-17^ direct analysis of brain-derived tau oligomers could offer novel insights. Brain-derived tau oligomers from Alzheimer’s disease individuals (hereafter referred to as BDTOs), and recently also tau in cerebrospinal fluid, have been found to impede long-term potentiation (LTP), an essential calcium-dependent process in synaptic plasticity, underlying learning and memory.^18,19^ Tau pathology seems to be directly responsible of calcium dysregulation,^20^ and in addition, we previously reported that the calcineurin inhibitor Tacrolimus (FK506), a US Food and Drug Administration–approved immunosuppressant drug, is associated with reduced incidence of Alzheimer’s disease.^21,22^ Calcineurin is a calcium- and calmodulin-dependent protein phosphatase that plays a regulatory role in various cellular processes, including neuronal signalling and synaptic plasticity.^23^ Calcineurin is involved in the dephosphorylation of tau protein, aiding in maintaining the balance between tau phosphorylation and dephosphorylation.^24,25^ This deep integration in crucial neuronal processes suggests its potential implication in a variety of neurological disorders.^26,27^ Based on these premises, we here hypothesized that inhibition of calcineurin prevents synaptic impairment caused by BDTO. We evaluated the potential neuroprotective properties of FK506, using electrophysiological and RNA-sequencing (RNA-seq) combined approach. Our study revealed that FK506 effectively counteracts synaptic impairment caused by BDTO effects, preserving transcription of key synaptic RNAs post-LTP, which is pivotal for the conversion of short- to long-term memory.

## Materials and methods

### BDTO isolation from human tissue

BDTO isolation was performed as previously described.^28^ This preparation generates tau enriched for BDTO, which we will term as BDTO. Briefly, 100 mg of fresh-frozen hippocampus tissues of a single Alzheimer’s disease case (female, 90 years old, Braak stage 6 and PMI 15 h) provided by The University of Michigan Brain Bank, neuropathological assessment conformed to the National Institute on Aging/Reagan Institute consensus criteria. The brain sample was homogenized in phosphate-buffered saline (PBS; Corning #46-013-CM)–containing protease (Roche, #11836153001) and phosphatase inhibitors (Roche, #490683700). Following homogenization, the tissues were centrifuged using a microcentrifuge to remove large debris. Tau oligomers were immunoprecipitated from this PBS soluble fraction by using the Pierce co-immunoprecipitation kit (Thermo Fisher #26149), with 75 µg of the T18 antibody, which has been demonstrated to selectively bind tau oligomers in the previous studies.^29-31^ To ensure the specificity of the T18 antibody, a mock pull-down using rabbit IgG isotype control was performed as a control, and the PBS soluble fraction was used as input for immunoprecipitation (Supplementary Fig. 1). The antibody–antigen complex was eluted using 0.1 M glycine buffer (pH 2.8) and neutralized with 1.0 M Tris-Cl (pH 8.0). The co-immunoprecipitation process was carried out under a fume hood, and samples were periodically agitated using an orbital shaker to maximize antibody–antigen interaction. Western blot analysis was performed to validate BDTO immunoprecipitation upon protein quantification with the Micro BCA kit (Thermo Fisher #23235). In particular, 5 µg of input (PBS soluble fraction), 10 µl of immunoprecipitated material was loaded on precast NuPAGE 4–12% Bis–Tris gels (Invitrogen) for sodium dodecyl sulphate–polyacrylamide gel electrophoresis analysis. Gel was subsequently transferred onto nitrocellulose membranes and blocked for 1 h at room temperature using Odyssey Blocking Buffer. The membrane was probed overnight with Tau 13 (1:20 000) at 4°C, as previously described.^31^ The following day, the membranes were washed and probed with IRdye secondaries antibodies (LI-COR) at 1:10 000 for 1 h at room temperature. The images were acquired by LI-COR Odyssey imager. Post-immunoprecipitation, the BDTOs from the patient with Alzheimer’s disease were amplified by using recombinant Tau 4R monomers (TauM), as previously described.^28,29,32,33^ Briefly, BDTOs were seeded with recombinant TauMs at a ratio of 1:100 (BDTOs:TauM, w/w), with a minimum seeding amount of 100 µg TauM per seeding event. The seeding process involved the use of low-retention pipette tips and tubes, with samples stored in 50 ml falcon tubes and regularly agitated using an orbital shaker for 2 days at room temperature. The obtained amplified BDTOs were used for further experiments.

### Animals, slice preparation and solutions

Male C57Bl/6 mice (*n* = 17, age = 8–12 weeks) were procured from Jackson Laboratories to conduct the experimental procedures. The animals were accommodated in filter-topped cages, with a maximum of four mice per cage, in a facility maintaining a stable environment at 22°C and 40% humidity, under a 12:12 h light–dark cycle. Standard chow and water were available *ad libitum*. Specifically, male mice of an age known to yield stable and robust synaptic plasticity experiments to manage sex and age variables were chosen. This acknowledges the variability in responses to Alzheimer’s disease proteins and treatments across genders and ages, narrowing the generalizability of our results but establishing a foundation for broader, more inclusive future research. Experimental groups comprised a minimum of three mice each, and a total of at least 12 hippocampal slices were analysed per group. However, slices exhibiting suboptimal field excitatory post-synaptic potential (fEPSP) recordings (characterized by unclear or indistinguishable signals with amplitudes <50µV) were systematically excluded from the analysis (CTRL *n* = 11; BDTO *n* = 7; BDTO + FK506 *n* = 8; FK506 *n* = 11). The expected effect size was estimated from preliminary data obtained from similar studies. For randomization, brain slices from each animal were allocated across different experimental groups using a random number generator to ensure balanced representation and minimize intra-animal variability. This process ensured unbiased allocation not influenced by prior knowledge or expectations. To further minimize potential confounders, several strategies were employed: the order of treatments and measurements for each animal was randomized to prevent systematic bias; environmental conditions such as temperature, humidity and lighting were kept constant; and the experimenter conducting the measurements and analyses was blinded to the treatment groups. These measures were taken to ensure that the results obtained are as reliable and replicable as possible. This study received approval from the Institutional Animal Care and Use Committee at the University of Texas Medical Branch (UTMB, Galveston, TX, USA) and was conducted in strict adherence to the National Institutes of Health (NIH) guidelines on the ethical use of laboratory animals.^34^ All methodologies and experimental procedures were performed in compliance with UTMB and NIH-approved guidelines and regulations. All the solution used in these experiments were constantly bubbled with 5% CO_2_ and 95% O_2_. The mice were euthanized under deep isoflurane anaesthesia and perfused using ice-cold NMDG-aCSF (N-Methyl-D-Glucamine artificial cerebrospinal fluid) solution (in mM: KCl 2.5; NaH_2_PO_4_ 1.2; NaHCO_3_ 30; HEPES (4-(2-Hydroxyethyl)-1-peperazineethanesulfonic acid) 20; glucose 25; sodium ascorbate 5; thiourea 2; sodium pyruvate 3; *N*-acetyl-cysteine 12; NMDG 92; CaCl_2_ 2; MgSO_4_ 2). Brains were surgically removed from the skulls and sliced using the Compresstome VF-300 (Precisionary Instruments, Greenville, NC, USA) in ice-cold NMDG-aCSF to obtain 350 µm thick brain slices. Slices were allowed to recover for 20 min in NMDG-aCSF at 35°C. Slices were then stored for at least 2 h in a recovery chamber containing holding-aCSF (in mM: KCl 2.5; NaH_2_PO_4_ 1.2; NaHCO_3_ 30; HEPES 20; glucose 25; sodium ascorbate 5; thiourea 2; sodium pyruvate 3; *N*-acetyl-cysteine 12; NaCl 92; CaCl_2_ 2; MgSO_4_ 2) solution at room temperature. Prior to recording, all slices were incubated an additional hour with regular holding solution, or holding solution + BDTO 100 nM. During electrophysiological recordings, the slices were perfused at 2.5 ml/min with regular aCSF (in mM: NaCl 124; KCl 2.5; CaCl_2_ 2; MgSO_4_ 2; NaH_2_PO_4_ 1.2; NaHCO_3_ 24; HEPES 5; glucose 13) or aCSF + FK506 10 µM, at 21 ± 1°C. FK506 (Prograf®) was obtained from UTMB pharmacy and diluted before each experiment in aCSF to reach final concentration of 10 µM.

### Electrophysiological recording

The fEPSP was evoked stimulating Schaffer collateral pathway via a concentric bipolar electrode (FHC Inc., Bowdoin, ME, USA—Cat# 30200), and recording electrode was located at the stratum radiatum (CA1). Recordings were digitized with Digidata 1550B (Molecular Devices, Sunnyvale, CA, USA), collected using an Axon MultiClamp 700B differential amplifier (Molecular Devices) and analysed using Clampex 10.6 software (Molecular Devices). Current stimulation was delivered through a digital stimulus isolation amplifier (AMPI, Israel). We performed an input–output protocol with stimulus ranging from 100 to 1000 mA (Supplementary Fig. 2), and for synaptic potentiation experiments, the stimulus was set to elicit an fEPSP ∼30% of maximum response. The online data acquisition software was used to monitor fEPSP slope every 20 s and average every three responses. After a stable baseline of 20 min, high-frequency stimulation (HFS), consisting of three trains of 100 pulses at 100 Hz with a 10-s interval, was used to induce LTP. LTP magnitude was measured as the average of fEPSP slope between the 50th and 60th minutes after HFS. The values were expressed as mean ± standard error of the mean (SEM) percentage change relative to their mean baseline fEPSP slope. For testing synaptic facilitation, two paired pulses (PPs) were administered within a 50-ms interval, which has been identified as optimal for eliciting substantial synaptic facilitation under experimental conditions similar to ours.^35-38^ PP facilitation was quantified by calculating the ratio of the synaptic response amplitude elicited by the second stimulus to that of the first stimulus, with the result expressed as a percentage. The order in which the protocols were performed is the following: (i) I/O; (ii) PP; (iii) baseline; (iv) HFS; and (v) post-HFS.

### RNA-sequencing

After completion of the electrophysiological experiments, hippocampi were meticulously isolated from the rest of the brain slice. The isolated tissues were immediately snap frozen in liquid nitrogen to preserve RNA integrity. RNA extraction was subsequently performed using the Monarch Total RNA Miniprep Kit (New England Biolabs). To ensure complete removal of potential DNA contamination, the optional DNAse1 digestion step was incorporated, as per the manufacturer’s instructions. The RNA samples were then used for sequencing library preparation following the Smart-3SEQ protocol. Briefly, 3′-end RNA-seq libraries were generated to specifically capture and sequence the 3′ ends of polyadenylated RNA. This approach enhances the quantification accuracy of gene expression levels by focusing on a consistent region across different RNA molecules. All sequencing experiments and subsequent quality checks were conducted at the Next Generation Sequencing core facility of the UTMB, ensuring standardized protocols and optimal conditions were met throughout the process. Libraries were prepared via the Smart-3SEQ method. This entailed annealing the first strand primer to the sample RNA and extending it using SMARTScribe reverse transcriptase from Clontech. Subsequent synthesis of the second strand was done post the addition of the primer, followed by polymerase chain reaction (PCR)-mediated integration of adapter sequences with unique indices utilizing NEBNext single-index adapters from New England BioLabs. These PCR products were then purified using AMPure XP SPRI beads and sequenced on the Illumina NextSeq 550 platform. In the sequencing reads, the distinctive five-base molecular identifier and three guanine bases incorporated by the Smart-3SEQ were eliminated, and the identifier was appended to the read name using the umi_homopolymer.py tool. These reads were mapped onto the *Mesocricetus auratus* NCBI genome assembly, and gene read counts were calculated using FeatureCounts, which fed into DESeq2 for differential gene expression analysis. Analysis of differentially expressed genes (DEGs) was done using JMP.software and Metascape for gene enrichment pathway analysis.

### Statistical analysis

**Figure 1:**
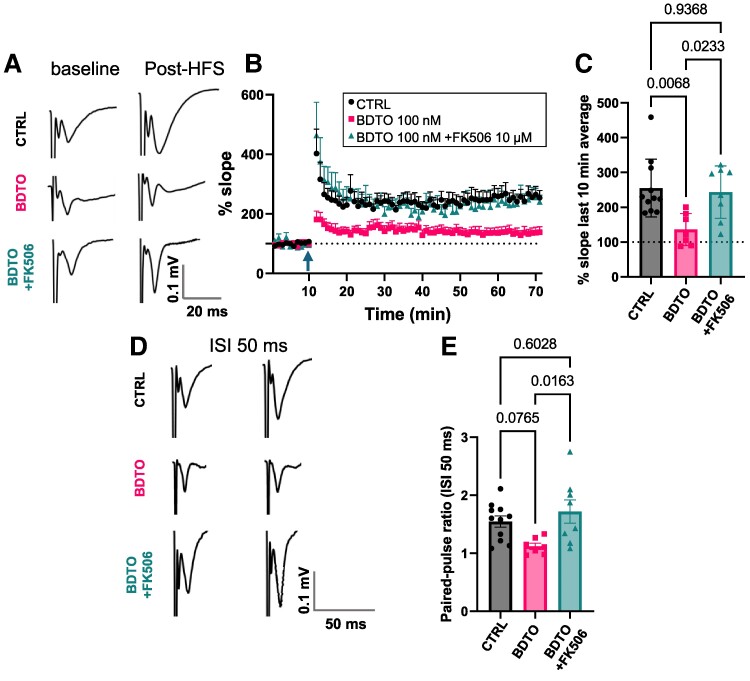
**FK506 prevents hippocampal synaptic plasticity impairment induced by BDTO.** Hippocampal slices from C57BL/6J mice (age: 8–12 weeks) underwent a 1-h pre-treatment with either standard holding solution or holding solution + BDTO (100 µM) prior to electrophysiological recording. Slices exposed to BDTO were then recorded using regular aCSF or aCSF + FK506 (10 µM). Field recordings were conducted with the stimulation electrode in the Schaffer collateral and the recording electrode in the Stratum Radiata of CA1 pyramidal neurons. Stimulation intensity was set to evoke 30% of maximal response. (**A**) Representative current traces of LTP protocol, average of 30 sweeps for baseline (10 minutes) and 180 sweeps post-HFS (60 min). (**B**) Slope percentage of fEPSP. (**C**) Slope average over the final 10 min showing FK506’s prevention of LTP impairment by BDTO (half-brain slices each group: CTRL *n* = 11; BDTO *n* = 7; BDTO + FK506 *n* = 8). (**D**) Representative current traces of PP stimulation protocol, average on 10 sweeps over 3 min, interstimulus interval (ISI) was 50 ms. (**E**) PP ratio indicating a trend towards impairment in the BDTO group compared with control and a significant reduction versus BDTO + FK506. Minimum of three different mice each group, each point represents data from a single half-brain slice (CTRL *n* = 11; BDTO *n* = 7; BDTO + FK506 *n* = 8, see the Statistical analysis section for criteria of exclusion). Statistical analysis involved one-way ANOVA followed by Tukey’s *post hoc* test.

**Figure 2:**
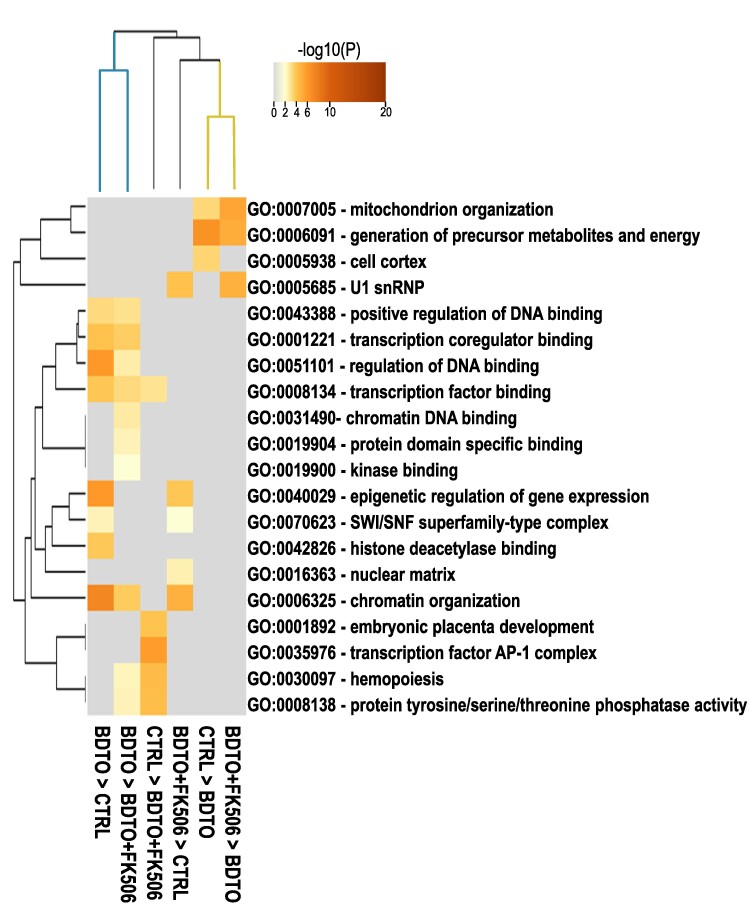
**Heat map illustrating PEA of DEGs in CTRL, BDTO and BDTO + FK506 groups.** DEGs were identified using an FDR *P*-value threshold in pairwise comparisons (e.g. CTRL > BDTO refers to genes upregulated in CTRL compared with BDTO; BDTO > CTRL refers to genes upregulated in BDTO compared with CTRL). Enrichment analysis involved accumulative hypergeometric *P*-values and enrichment factors (gene ontology terms). A hierarchical clustering approach was employed to categorize experimental groups. The heat map’s value scale ranges from 0 (indicating non-significance) to 20 (representing statistical significance in gene expression variations between groups). The analysis highlights specific pathways and gene expressions modulated differently across treatment conditions (CTRL *n* = 11; BDTO *n* = 7; BDTO + FK506 *n* = 7, see the Statistical analysis section for criteria of exclusion). U1 snRNP, U1 small nuclear ribonucleoprotein; SWI/SNF, switch/sucrose non-fermentable; AP-1, activator protein 1.

The sample size for each experimental group was determined based on a power analysis, aiming to ensure sufficient statistical power to detect a significant difference between groups with an alpha level of 0.05 and a power of 80%. Six slices in each group were considered the minimum for having sample size = 0.6. All data are expressed as mean ± SEM. In Fig. 1 and Supplementary Fig. 3, we used one-way ANOVA followed by Tukey’s *post hoc* test. In Fig. 2, we employed a one-way ANOVA to assess statistical differences. To address the issue of multiple comparisons and control the false discovery rate (FDR), we applied an FDR-adjusted *P*-value approach. This method was utilized to discern practical differences among the groups. We discard one BDTO + FK506 slice because the sequencing did not pass the quality standard of a minimum read depth and uniformity across the targeted regions, as well as having a high percentage of low-quality base calls. Supplementary Fig. 2 was used two-way ANOVA multiple comparison. The stimulus-response curve was fit using third order polynomial model equation: Y = B0 + B1 × X + B2 × X^2^ + B3 × X^3^.

## Results

### Electrophysiology

To test the hypothesis that calcineurin inhibition prevents synaptic impairment caused by BDTO in regions of the brain that are vulnerable to Alzheimer’s disease pathology, we performed field recording experiments in the hippocampal Shaffer collateral—CA1 synaptic pathway of wild-type C67BL/6J mice. Prior to conducting the electrophysiological recordings, we incubated hippocampal slices in a small chamber with 450 µl of oxygenated holding solution either with or without BDTO for 1 h. Previous research has demonstrated that this duration of incubation is adequate for primary neurons to internalize BDTO.^33^ Then, slices incubated with BDTO were recorded in the presence or absence of FK506. Basal synaptic transmission did not show differences within the experimental groups (Supplementary Fig. 2). We observed that the treatment with BDTO repressed LTP by decreasing fEPSP slopes (Fig. 1A–C), and this toxic effect was blocked if the recording was done in the presence of FK506 [one-way ANOVA, *F*(2, 23) = 6.376, *P* = 0.006 followed by Tukey’s multiple comparisons]. To determine dynamic properties of synaptic transmission, specifically short-term plasticity, we performed PP stimulation (Fig. 1D and E). BDTO caused a trend of decrease in the second response of the PP compared with CTRL group, that ultimately was re-established in the presence of FK506 [one-way ANOVA, *F*(2, 23) = 4.793, *P* = 0.018, followed by Tukey’s multiple comparisons]. To exclude any effect of FK506 alone, we compared control slices either in the presence or in the absence of FK506 (Supplementary Fig. 3), and we did not find any differences in fEPSP (unpaired *t*-test *P* = 0.356) and PP (unpaired *t*-test *P* = 0.813).

### RNA-sequencing

To further explore whether BDTO and FK506 treatments together with HFS produced specific transcriptomic changes, we investigated transcripts levels on the same hippocampal slices subjected to HFS and field recording (LTP protocol). After the electrophysiological experiments, we isolated the hippocampus and snap froze the slices (∼4 h after incubation and 3 h after HFS). We first evaluated the effect of HFS on CTRL slices by comparing stimulated with no stimulated slices. We performed multiple comparison tests specifically applying an FDR *P*-value for the practical difference test. We found 387 DEG between CTRL stimulated and CTRL no stimulated. Notably, most of such genes were related with regulation of gene expression and chromatin remodelling (Supplementary Fig. 4 and Table 1), suggesting massive changes in transcription regulation after HFS. We then screened differences in mRNAs levels among the experimental groups that received HFS: CTRL (stimulated), BDTO and BDTO + FK506 (Supplementary Table 2). We found 275 DEG between CTRL and BDTO groups (158 higher in CTRL + 117 higher in BDTO), 288 DEG between BDTO and BDTO + FK506 groups (136 higher in BDTO + 152 higher in BDTO + FK506) and 179 DEG between CTRL and BDTO + FK506 (95 higher in CTRL + 84 higher in BDTO + FK506). We then performed pathway enrichment analysis (PEA) of each of the DEG groups (Fig. 2). In order to improve our comprehension of the affected pathways and to facilitate the comparison of the six gene lists, we conducted a hierarchical cluster analysis. The results of this analysis are presented as a clustered heat map accompanied by a dendrogram containing multiple branches (Fig. 2). The proximity of these branches indicates a higher degree of shared pathways among the lists. Two of them showed PEA in both CTRL and BDTO + FK506 versus BDTO, and they contained pathways related to mitochondria, energy, regulation of DNA and transcription. The other two branches showed differences between CTRL and BDTO + FK506 groups and they contained pathways related to transcription factor activator protein 1 (AP-1) complex, protein tyrosine/serine/threonine phosphatase activity and DNA regulation. This result showed that BDTO + FK506 was better associated with CTRL then BDTO, suggesting that FK506 treatment was able to restore some of the key mRNAs that were impaired by BDTO.

To test the potential modulation of inflammatory genes by BDTO and FK506 treatment, we selected genes that positively (GO:0050729) or negatively (GO:0050728) influence the inflammatory cascade. DEG analysis was conducted through pairwise comparisons, mirroring the approach used in Fig. 2. Specifically, we compared: CTRL versus BDTO, with CTRL upregulated genes (CTRL > BDTO) and downregulated genes (CTRL < BDTO); BDTO versus BDTO + FK506, with BDTO upregulated genes (BDTO > BDTO + FK506) and downregulated genes (BDTO < BDTO + FK506); and BDTO + FK506 versus CTRL, with upregulated genes (BDTO + FK506 > CTRL) and downregulated genes (BDTO + FK506 < CTRL). The quantity of genes modulating inflammation did not show marked variations within the groups studied. Nonetheless, a comprehensive list of DEG was compiled for each pairwise comparison in Supplementary Table 3. Specifically, in comparison with the BDTO + FK506 group, the CTRL group exhibited an upregulation of 10 pro-inflammatory genes, including *H2-M5*, known for its role in antigen presentation [1], [2] and *Pla2g3*, implicated in the biosynthesis of pro-inflammatory eicosanoids [3]. Additionally, CTRL presented an upregulation of 14 anti-inflammatory genes such as *Foxp3*, a transcription factor critical for the regulatory T-cell function [4], [5] and *Nod2*, known for its role in the negative regulation of immune responses [6] and [7].

For the BDTO + FK506 group compared with CTRL, genes such as *Pdcd4*, involved in apoptosis and inflammation [8], and *Clock*, associated with circadian regulation of immune responses [9], were upregulated. In the comparison between BDTO and BDTO + FK506, genes such as *Ripk1*, a key regulator of inflammation and cell death [10], and *Tnf*, a major pro-inflammatory cytokine [11], were more expressed in BDTO.

In the anti-inflammatory gene set, BDTO + FK506 showed higher expression levels of *Cx3cr1*, which encodes a receptor involved in the negative regulation of leucocyte adhesion and migration [12], [13] and *Cd276*, which has been implicated in the suppression of T-cell activation [14]. Conversely, BDTO displayed elevated levels of *Ash1 l*, know to its role in chromatin modification and suppression of the IL-6 [15] and *Bcr* crucial for the maturation, antigen-specific growth and survival of B cells [16], compared with CTRL.

### LTP correlates with synaptic transcripts

**Figure 3:**
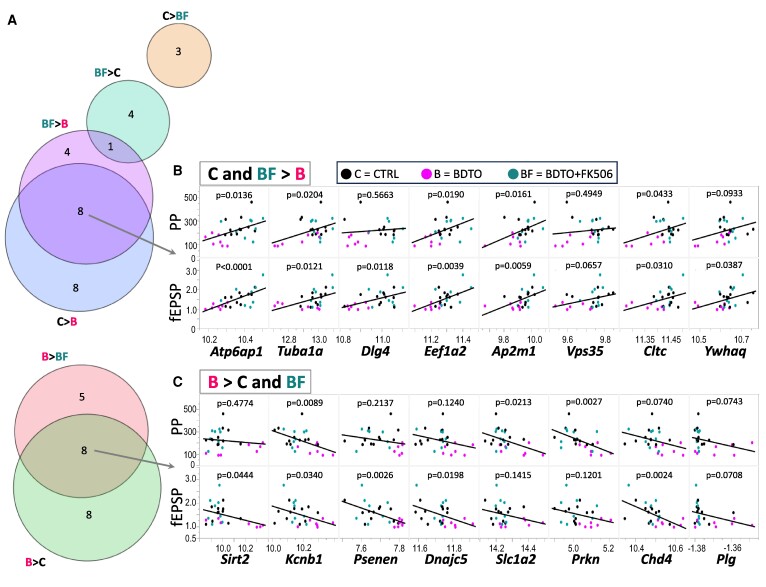
**Synaptic gene expression and correlation with electrophysiological parameters.** (**A**) Venn diagram illustrating the overlap and uniqueness of DEGs across different treatment groups. (**B**, **C**) Pearson’s correlation analysis between synaptic DEGs and electrophysiological measurements from Fig. 1 (LTP and PP ratios). Genes upregulated in CTRL and BDTO + FK506 groups compared with BDTO (**C**) show a positive correlation with electrophysiological measurements, whereas genes upregulated in BDTO compared with CTRL and BDTO + FK506 (**D**) demonstrate a negative correlation. The data suggest a distinct gene expression profile in the BDTO group that inversely correlates with synaptic plasticity measures. The list of the genes in Supplementary Table 4.

To elucidate the impact of BDTO on synaptic mRNAs, and whether FK506 was able to counteract their effect, using the same hippocampal slices, we focused our attention specifically on 1112 genes relevant to synaptic functioning as identified by the SYNGO database.^39^ Our approach employed a multiple comparison test (with FDR *P*-value for the practical difference test), mirroring the method in Fig. 2. Synaptic DEGs were represented using a Venn diagram (Fig. 3B). We observed that 40% of the genes that were upregulated (*Atp6ap1*, *Tuba1a*, *Dlg4*, *Eef1a2*, *Ap2m1*, *Vps35*, *Cltc* and *Ywhaq*) or downregulated (*Sirt2*, *Kcnb1*, *Psenen*, *Dnajc5*, *Slc1a2*, *Prkn*, *Chd4* and *Plg*) in the CTRL group compared with the BDTO group also showed a similar trend in the BDTO + FK506 group when compared with the BDTO group (Fig. 3A and B). These genes seem to be influenced by BDTO, but their expression is rectified by FK506. In addition, we found nine genes modulated uniquely by FK506 compared with BDTO (upregulated: *Elavl4*, *Chrm3*, *Rpl23a* and *Snap91*; downregulated: *Adgrb1*, *Prkar2a*, *Rps23*, *Rpl38* and *Rps27*), and 16 genes modulated specifically by BDTO compared with CTRL (upregulated: *C1ql3*, *Grin2b*, *Bcr*, *Psen1*, *Sipa1l1*, *Pde4a*, *Arhgef7* and *Syt11*; downregulated: *Rab8a*, *Cdc42*, *Snapin*, *Rab5b*, *Vamp4*, *Eif2s1*, *Eps15* and *Cacng7*). Interestingly, most of the genes dysregulated by BDTO and restored by FK506 were correlated with LTP, as shown by Pearson’s correlations between gene expressions and both fEPSP and PP (Fig. 3C and D). This result suggests that FK506 treatment can restore not only some of the basic plasticity mechanism in hippocampus, but also some of the early LTP downstream synaptic transcripts.

## Discussion

By employing a combination of electrophysiological and mRNA-seq techniques, our investigation provides evidence of the central role of calcineurin inhibition in mitigating the synaptic dysfunctions caused by BDTO, particularly in the hippocampus, a highly vulnerable brain region to Alzheimer’s disease pathology. This outcome aligns with our prior research, underscoring the diminished prevalence of Alzheimer’s disease and dementia in individuals prescribed the calcineurin inhibitor tacrolimus (FK506), thereby suggesting its protective properties.^21,22^

Using wild-type mice, we performed extracellular field recording of hippocampal Shaffer collateral—CA1 synaptic pathway confirming prior findings by others that BDTO hampers LTP by diminishing fEPSP slopes.^18^ This detrimental effect was effectively neutralized in the presence of FK506. Similarly, when we tested short-term potentiation, we found that BDTO decreased the ability of the synaptic transmission facilitation, re-established when in the presence of FK506. PP facilitation primarily indicates an augmented neurotransmitter release probability following the second pulse, likely attributable to residual Ca^2+^ in the pre-synaptic terminal from the first pulse. However, synaptic transmission involves complex interactions between pre- and post-synaptic elements, and in some contexts, changes in post-synaptic sensitivity can also influence the overall synaptic response.^40,41^ To examine whether basal EPSPs varied across groups, we generated input–output curves. These curves revealed no significant differences among the three groups (Supplementary Fig. 2). Consequently, the diminished PP facilitation observed in BDTO does not appear to result from an inherently higher neurotransmitter release probability during the first pulse. We also tested FK506 alone that showed no differences in fEPSP slope and PP when compared with the CTRL group (Supplementary Fig. 3) excluding any effect on synaptic plasticity of FK506 alone at the concentration used (10 µM). Guided by our latest findings, we tailored the subsequent transcriptomic analysis to primarily assess FK506’s capacity to neutralize the harmful impact of BDTO, focusing on three experimental groups: CTRL, BDTO and BDTO + FK506. Although our electrophysiological data did not indicate significant effects of FK506 alone on synaptic plasticity and existing literature lacks evidence this does not comprehensively rule out the drug’s potential transcriptomic impacts when administered independently.

Considering that toxic tau induces rapid structural changes in neuronal nuclei,^42,43^ which in turn can lead to chromatin rearrangements affecting transcription,^44-46^ and that memory consolidation necessitates the synthesis of *de novo* mRNA,^47,48^ we conducted mRNA-seq on the identical hippocampal slices employed in our electrophysiology experiments. Our initial focus was to determine the sole impact of HFS by comparing the gene expression in CTRL stimulated with HFS versus CTRL no stimulated. In line with expectations for LTP responses, genes, such as *Arc* and *Sgk1*, were significantly elevated in the HFS-exposed CTRL group compared with the non-stimulated CTRL group. *Arc* is known to influence synaptic strength and reorganize the actin cytoskeleton post-LTP,^49^ while *Sgk1* plays a role in the modulation of ion channels, neurotransmitter receptors and various signalling pathways.^50^ Interestingly, other canonical LTP-responsive genes, such as *CREB*, *BDNF* and *c-fos*, did not show differential expression following HFS, which might be due to the normalization of their expression levels within the 3-h post-induction timeframe and the fact that our samples comprise a heterogeneous mix of cells, not exclusively neurons. Broadening our analysis, enrichment pathway analysis of all DEG unravelled pronounced alterations between CTRL stimulated with HFS versus CTRL no stimulated, predominantly linked to gene regulation and chromatin remodelling. Furthermore, we compared the three HFS-stimulated experimental groups (CTRL, BDTO and BDTO + FK506), our transcriptomic analysis highlighted a closer similarity between BDTO + FK506 with CTRL, diverging notably from the BDTO-alone group. The hierarchical clustering shows this distinction, where two of the major branches of the dendrogram highlighted differences in mitochondrial genes, energy pathways and DNA transcription regulation. This evidence suggests that FK506 aids the preservation of mitochondrial integrity and function, helping in counteracting the energy deficits and oxidative stress caused by the impaired mitochondrial activity, common in tauopathies.^51^ Additionally, FK506 showed efficacy in modulating the general cellular energy metabolism, further contributing to the restoration of cellular homeostasis initially perturbed by the BDTO. To address the issue of sample cell type heterogeneity, we narrowed our focus to the impact of BDTO on synaptic mRNAs (neuronal) and its possible rectification by FK506. From the examination of 1112 genes pivotal for synaptic functioning, as delineated by the SYNGO database, we identified 49 DEG across the 3 groups, observing that FK506 demonstrated promising restorative effects on ∼40% of the genes dysregulated by BDTO. This group includes key players, such as *Atp6ap1*, *Dlg4* (PSD-95), *Ywhaq* (14-3-3 protein theta), *Slc1a2* (GLT-1), *Psen1* and *Prkn*. Delving into these specific genes provides critical molecular insights into FK506’s protective mechanisms on synaptic plasticity within the intricate framework of Alzheimer’s disease pathophysiology. *Atp6ap1* is implicated in the endosomal–lysosomal system, a pathway increasingly recognized for its role in Alzheimer’s disease–associated neurodegenerative processes.^52^  *Dlg4*, also referred to as *PSD-95*, is integral to post-synaptic density structures and is essential for maintaining synaptic plasticity.^53,54^ Previous research has linked aberrant *Dlg4* activity to synaptic deficits characteristic of Alzheimer’s disease.^55^ Concurrently, *Ywhaq*, known as the 14-3-3 protein theta, interacts directly with phosphorylated tau,^56,57^ and it is downregulated in Alzheimer’s disease.^58^ The *Slc1a2* gene, denoted as *GLT-1*, is central to the modulation of glutamate uptake, and alteration in its expression is related with sporadic Alzheimer’s disease.^59,60^ Presenilin genes, included *Psen1*, are central to certain familial Alzheimer’s disease forms and are integral for amyloid precursor protein processing and the regulation of intracellular calcium dynamics.^61,62^ The *Prkn* gene is known for its role in Parkinson’s disease due to its involvement in mitophagy and mitochondrial quality control.^63^ Although not a classic genetic marker for Alzheimer’s disease, *Prkn*’s role in cellular maintenance, including mitochondrial regulation and autophagy, may intersect with Alzheimer’s disease mechanisms, indicating potential relevance to the mitochondrial dysregulation observed in Alzheimer’s disease pathology.

This intricate genomic modulation appears consistent with the observed electrophysiological changes. Many genes disturbed by BDTO, and subsequently normalized by FK506, exhibit significant correlations with PP and fEPSP. This interplay suggests that changes in mRNA expression related to synaptic functions are closely associated with alterations in synaptic plasticity. Furthermore, these observations may reveal novel molecular targets and mechanisms by which FK506 preserves synaptic function against the detrimental effects of BDTO.

Some of the genes were modulated by BDTO but not restored by FK506, including *Chrm3*, which encodes the muscarinic acetylcholine receptor M3, known for its involvement in cognitive functions.^64^ Additionally, a subset of genes was uniquely modulated by the BDTO + FK506 compared with BDTO alone, indicating a unique modulation of the FK506 on the toxic environment induced by BDTO treatment. Among these, *Grin2b* and *Psen1* stand out for their direct relevance to this study. *Grin2b* is crucial for synaptic plasticity.^65^ Psen1 mutations are a known genetic cause of Alzheimer’s disease^61^ and reduction of calcineurin activity in presenilin 1 M146V mutant rescue physiological synaptic plasticity.^66^

Application of BDTO or BDTO in combination with FK506 in our study did not result in a massive variation in the expression of genes modulating inflammation across the different groups. Notably, in the CTRL group compared with the BDTO + FK506 group, there was an upregulation of small number of pro-inflammatory and anti-inflammatory genes, indicating a nuanced effect of FK506 on inflammatory pathways. Moreover, our findings suggest that while FK506 influences the expression of some genes related to inflammation and immune response, the overall impact on inflammatory gene expression appears to be limited in the context of our experimental conditions may due to the short time between treatments and storage of the brain slice at −80°C. However, prolonged administration of calcineurin inhibitor, as observed in previous studies,^21,22^ could potentially yield anti-inflammatory effects of FK506, leading to neuroprotection. This effect may be mediated by inhibiting the calcineurin/Nuclear Factor of Activated T-cells pathway implicated in the regulation of glutamate homeostasis through the downregulation of *Glt1/EAAT2*.^67,68^ Loss of *Glt1/EAAT2* can lead to neuronal hyperexcitability,^69^ which is one of the alteration found in Alzheimer’s disease.

In summary, our findings converge to strengthen the therapeutic promise of FK506 in addressing synaptic and transcriptional abnormalities that arise in the context of Alzheimer’s disease. These revelations serve as a guiding light, directing further in-depth investigations into the precise mechanisms of action of FK506 and its potential clinical implications.

## Supplementary Material

fcae277_Supplementary_Data

## Data Availability

The data supporting the findings of this study are available on request from the corresponding authors.
