# Supplementary material for: Calcineurin inhibition prevents synaptic plasticity deficit induced by brain-derived tau oligomers
Source: Brain Commun. 2024 Aug 16;6(5):fcae277. doi: 10.1093/braincomms/fcae277 (PMC11375858; doi:10.1093/braincomms/fcae277)
Supplement: fcae277_Supplementary_Data [file fcae277_supplementary_data.zip › Supplementary_Figures 1-4.pdf]

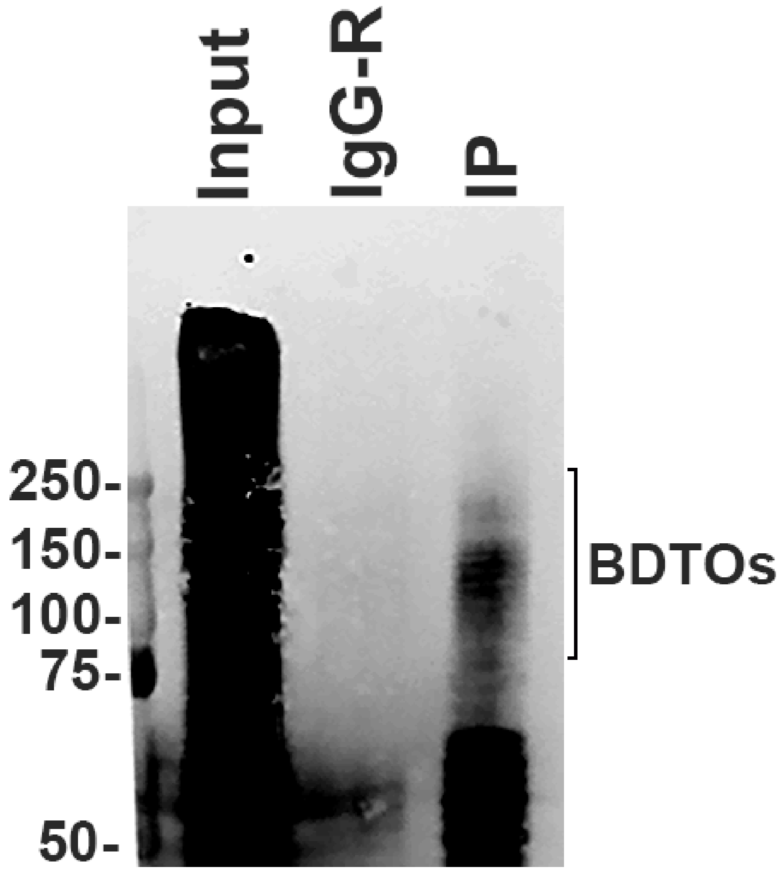

Supplementary Figure 1. Quality control of BDTOs immunoprecipitation. Representative immunolabeling of T18 immunoprecipitated (IP) BDTOs using Tau13 antibody showed high molecular weight tau oligomers in PBS-soluble fraction. No immunoreactivity to T13 was observed when the sample was immunoprecipitated using the IgG isotype control (IgG-R).

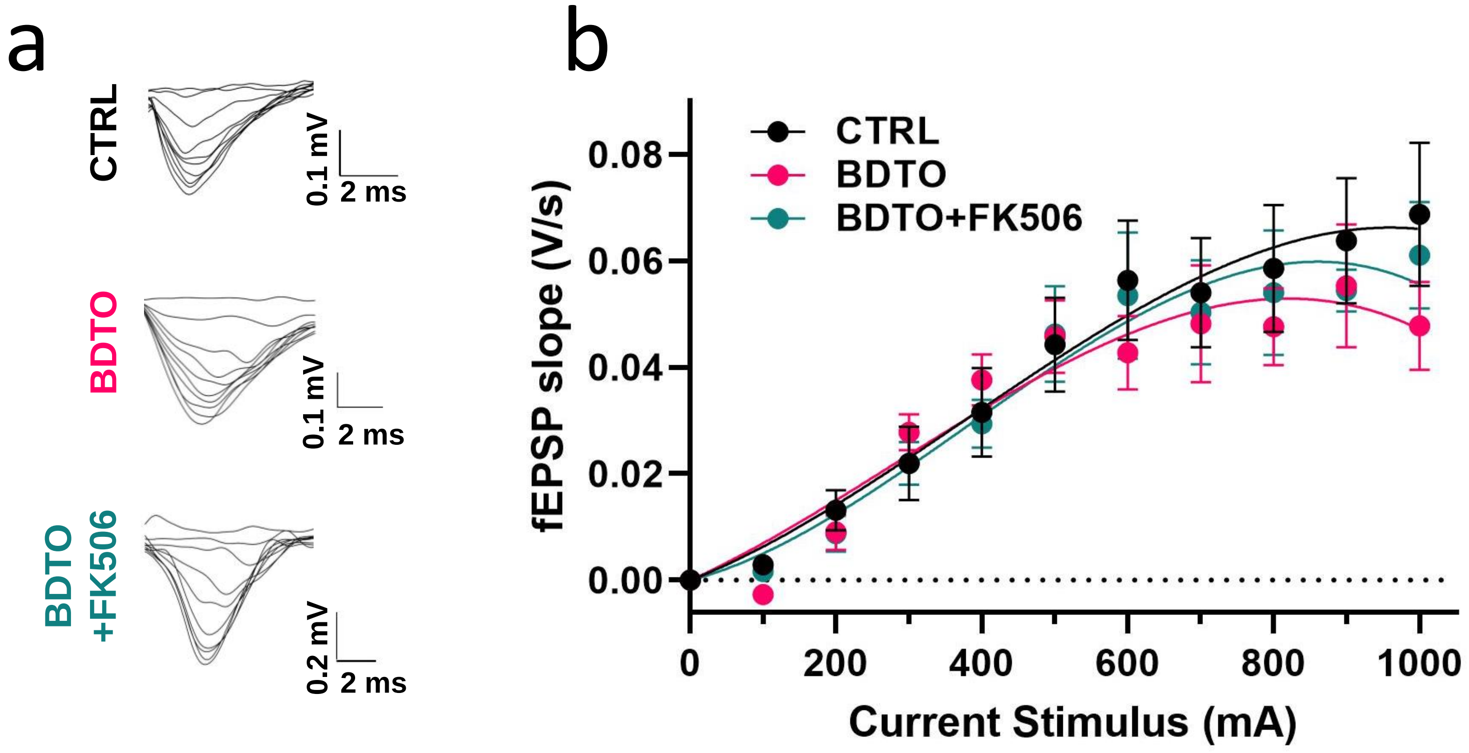

Supplementary Figure 2. Input output curve of CTRL, BDTO, and BDTO+FK506 group. Basal synaptic transmission at the CA3-CA1 connection in slices from 8-12 month-old wild type mice. Hippocampal slices were treated with vehicle or BDTO (recorded with or without FK506) a. Representative current traces of input-output protocol (100 to 1000 mA stimuli). b Basal synaptic transmission represented with fEPSP vs stimulus strength did not show differences across the three experimental groups using two-way ANOVA multiple comparison p=0.937, (N half-brain slices = CTRL 10, BDTO 6, BDTO+FK506 6, see statistical analysis paragraph for criteria of exclusion). The stimulus-response curves were fit using third order polynomial model equation: Y=B0 + B1*X + B2*X^2 + B3*X^3.

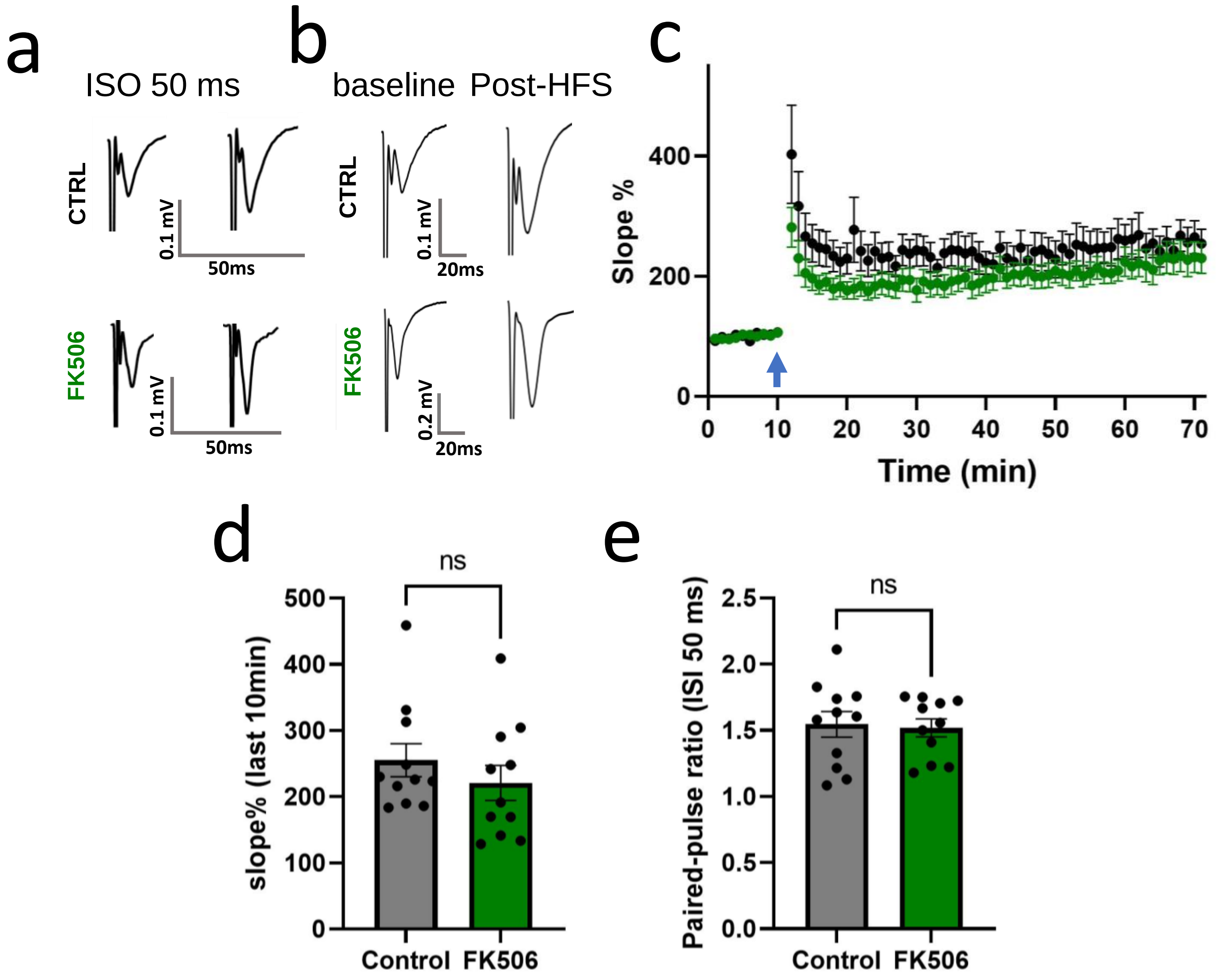

Supplementary Figure 3. FK506 alone does not affect synaptic plasticity. Electrophysiological field recording of hippocampal slices from 8-12 weeks old C67BL/6J. Stimulation electrode was placed in Shaffer collateral and recording electrode in stratum radiata of CA1 pyramidal neurons. Shaffer collateral were stimulated evoking the 30% of the maximal response. a Paired-pulse ratio using interstimulus interval (ISI) of 50ms. b Representative current traces of long-term potentiation (LTP) protocol. c fEPSP percentage of the slope. d Percentage slope average of the last 10min. e Paired-pulse ratio. Minimum of 3 different mice each group, each point is a single brain slice (CTRL n = 11; FK506 n = 11). For statistical test was used unpaired t-test: fEPSP (unpaired t test p = 0.356) and PP (unpaired t test p = 0.813).

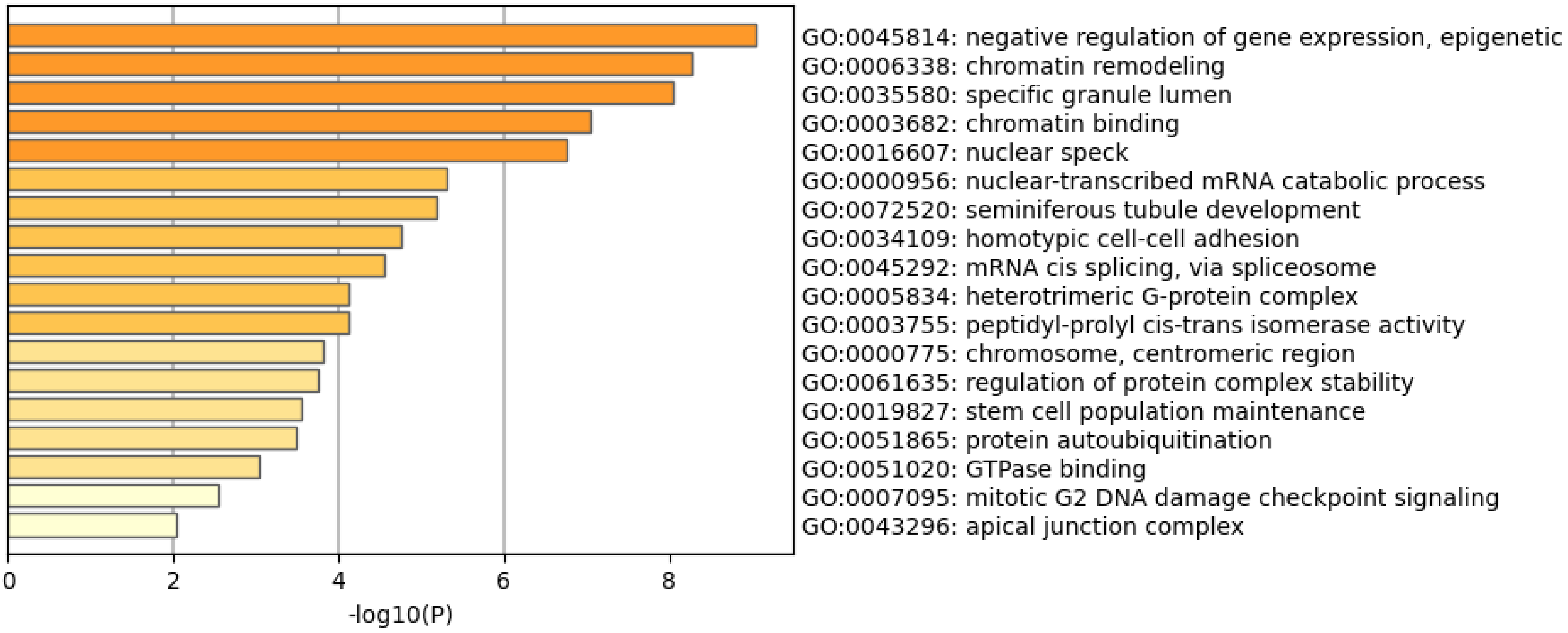

Supplementary Figure 4. Effect of High frequency stimulation (HFS) on RNA transcription in mouse hippocampus. Pathway enrichment analysis of differential expressed genes (DEG) in CTRL stimulated with HFS (N=11 slices) compared to the CTRL no stimulated (N=4 slices).
